# Identifying quality indicators for home care services: a modified Delphi and Analytic Hierarchy Process study

**DOI:** 10.1186/s12912-024-02169-4

**Published:** 2024-07-19

**Authors:** Qiu-Lan Zheng, Ling-Na Kong, Ping Hu, Dun-Xiu Liu

**Affiliations:** 1 Department of Nursing, The Second Affiliated Hospital of Chongqing Medical University, Chongqing, China; 2 School of Nursing, Chongqing Medical University, Chongqing, China; 3 Department of General Practice, The First Affiliated Hospital of Chongqing Medical University, Chongqing, China; 4 Department of Neurology, The First Affiliated Hospital of Chongqing Medical University, Chongqing, China

**Keywords:** Home care services, Quality indicators, Quality of care, Delphi, Analytic Hierarchy Process, Nursing management

## Abstract

**Background:**

As the recipients of home care services, patients have the most direct and profound experience of service quality. There is limited knowledge as to quality indicators for home care services from patients’ perspective. This study aimed to identify quality indicators for home care services based on the Service Quality model and determine the weights of these indicators.

**Methods:**

A two-round Delphi survey and Analytic Hierarchy Process consultation were conducted to gather opinions from national experts on quality indicators for home care services developed on the basis of the Service Quality model. Consensus was defined as at least 80% agreement on the importance (important and very important) of indicators among experts. The Analytic Hierarchy Process was used to calculate the weight coefficients of the identified indicators.

**Results:**

The response rate was 95.0% and 97.4% in the first and second round, respectively. After two rounds, five first-level (tangibility, reliability, responsiveness, assurance and empathy) and 23 second-level indicators were identified. The Kendall’s W values were 0.54 and 0.40 for the first-level and second-level indicators (*p* < 0.001). The weight coefficients for the first-level and second-level indicators were 0.110–0.298 and 0.019–0.088, respectively.

**Conclusion:**

Quality indicators for home care services were identified based on the Service Quality model. These indicators can be used to evaluate the service quality of home care from patients’ perspective and facilitate to determine work priorities and improve the quality of home care.

**Supplementary Information:**

The online version contains supplementary material available at 10.1186/s12912-024-02169-4.

## Background

In response to the growing aging population and increasing complexity in health problems, many countries tend to move nursing care from hospitals to home settings [1], including in China. Home care can provide assistance for people with various health care needs to live as independently as possible in their private homes [2]. Evidence have shown that good quality home care can improve patients’ activities of daily living and quality of life [3] and reduce hospital admission [4] and caregiver burden [5]. Home care services vary considerably in scope of service and eligibility requirements between countries and each country should provide home care services depending on its own guidelines [6].With the rapid development of home care, there are shared concerns on the care quality.

Due to the increasing burden of aging and relative lack of health service resources, the Chinese Health Committee has proposed developing community and home care services rapidly to satisfy people’s health needs, especially for older adults with chronic diseases [7]. In China, home care is defined as the nursing care and personal care for people of all ages provided by registered nurses in people’s private homes. Home care services are usually provided by registered nurses from community health centers and hospitals, and care recipients are mainly patients who need nursing care after discharge, older adults with chronic diseases and the disabled [8]. Despite the rapidly increasing need of home care, some existing problems may hinder the quality of care, such as shortage of home care nurses, considerable variations in nurses’ competence across home care institutions and lack of standardised home care rules, regulations, process and quality monitoring system [9]. Home care institutions are under growing pressure to offer good quality care.

Measurement of service quality plays an important role in ensuring that patients’ care needs are satisfied and identifying areas that required to be improved. Efforts have been undertaken to develop quality indicators for home care, such as the Outcome Assessment Information Set (OASIS) [10], Home Care Quality Indicators (HCQIs) [11] and Resident Assessment Instrument-Home Care system (RAI-HC) [12]. These indicators mainly focus on measuring patients’ functional status, basic background information on housing, needs for care and home care risks. As the recipients of home care services, patients have the most direct and profound feelings and experience of service quality. Measuring quality of care from patients’ perspective is being increasingly used in healthcare research [13]. Shaller et al. [14] have proposed that the quality improvement in health care should incorporate care recipients’ opinions. Thus, understanding patients’ evaluation of service quality of home care can give clear feedback to service providers to identify problems that need to be improved and facilitate actual improvements in quality of care.

The Service Quality (SERVQUAL) model has been widely used to measure service quality based on consumers’ perspective [15]. It covers five basic dimensions: tangibility (the appearance of the facilities and personnel), reliability (ability to provide the promised service accurately), responsiveness (promptness and helpfulness), assurance (competence, credibility and courtesy) and empathy (caring and individualised attention) [16]. These dimensions represent the key factors of service provision, however, they may not encompass all factors in all service settings [17]. Researchers have been suggested to modify the existing indicators or develop their own indicators to their socio-demographic, cultural and geographical context based on the SERVQUAL model [16]. Previous studies have measured service quality on the basis of the SERVQUAL model in public hospital [18], hospice care [19], outpatient teaching hospital pharmacies [20] and dental service [21].

Home environment influenced care or care strategies and quality of care provided by staff may be hindered by the home environment [22]. Patients are increasingly paying attention to the service quality of home care. There is a need to assess service quality of home care to understand what patients consider as “good care” and improve the care process and staff functioning. Service quality is highly culture-centric. There is limited knowledge as to quality indicators for home care services from patients’ perspective. Therefore, the objectives of this study were to establish quality indicators for home care services based on the SERVQUAL model and determine the weights of these indicators. The importance of these indicators lies in evaluating quality of home care services from patients’ perspective and providing information on areas that need to be improved.

## Methods

### Design

The Delphi technique was applied to reach consensus on quality indicators for home care services. The Delphi method can synthesise the knowledge of a group of experts and has been extensively used for the development of quality indicators in healthcare [23]. We used the modified Delphi technique which consists of beginning the process with a set of carefully selected items. The primary advantages of this modification were to typically improve the response rate, provide a solid foundation based on current evidence and control the feedback from experts. The Analytic Hierarchy Process (AHP) was used to determine the weights of quality indicators according to the opinions of a group of experts. AHP is a multi-criteria decision-making technique proposed by Satty and has been used in a variety of clinical decisions because it is methodologically sound and user-friendly [24].

### Experts panel

Using a purposive sampling method, we recruited national experts from the fields of home care management, practices and research. Potential experts were identified from extensive review of the literature or suggested by the members of the research group. The inclusion criteria of experts were as follows: (1) having a minimum of five years of experience in fields related to home care; (2) with a Bachelor’s degree or above; and (3) voluntarily participation in several rounds of consultation. We recruited 40 experts from 6 community health centers, 8 tertiary hospitals and 9 medical schools across 11 provinces (municipalities) in China.

### Questionnaire preparation

Based on the framework of the SERVQUAL model, five dimensions of quality indicators for home care services included tangibility, reliability, responsiveness, assurance and empathy. A list of potential indicators for the five dimensions was drafted by the following procedures. First, a literature review of quality indicators for healthcare service based on the SERVQUAL model was carried out to collect a list of quality indicators. Second, we conducted semi-structured interviews with home care administrators and home care recipients to further collect indicators. The questions were mainly on the key aspects of service quality for home care services, such as “what home care service is considered good?” and “which services were good and which were needed to be improved?”. Third, a group discussion by researchers was conducted to identify the potential quality indicators (five first-level and 30 second-level indicators) based on the literature review and semi-structured interviews. The first-level indicators refer to the five dimensions of service quality of home care. The second-level indicators refer to the indicators for each dimension: tangibility (6 indicators), reliability (7 indicators), responsiveness (6 indicators), assurance (6 indicators) and empathy (5 indicators). In home care settings, tangibility refers to the equipment, service description and appearance of staff in home care institutions. Reliability refers to the ability of home care nurses to perform the promised service dependably and accurately. Assurance refers to the knowledge and courtesy of home care nurses and their ability to inspire trust among patients. Responsiveness refers to the ability of home care nurses to provide timely service. Empathy refers to the caring and individualised attention from home care nurses.

The consultation questionnaire was developed based on the above potential quality indicators. It consisted of three sections: (1) preface including research background, objective and methods of the survey, questionnaire completion requirements, time of questionnaire recovery and contact information of the researcher; (2) main text including five first-level and 30 s-level indicators (Table S1), definition of the first-level indicators and free-text comments column for experts; and (3) demographic information of experts, such as age, gender, education level, years of experience, areas of expertise and institutions. To check the face validity of the questionnaire, we consulted a home care administrator, a home care nurse and a researcher specialised in home care regarding its wording and clarity.

### Data collection

The questionnaires were delivered to experts via e-mail. Experts were instructed to rate the importance of each indicator on a five-point Likert scale (1 = very unimportant to 5 = very important). The consensus levels usually ranged from 70 to 80% agreement on the higher-level expert ratings in studies on identifying quality indicators using the Delphi method [25, 26]. In this study, consensus was defined by at least 80% agreement on rating the indicator between 4 and 5 (important and very important). Experts were also invited to make free comments on each indicator and suggest the addition of specific indicators. It took about 20 min to complete the questionnaire. Each round was conducted within two weeks, with one month between the two rounds for data analysis and indicator refinement. One reminder was sent to experts during the second week of each round if needed. Experts who participated in the first-round survey were included in the second round. In round two, experts were given the results of round one including the mean score, standard deviations (SD) and consensus rating of each indicator. They were asked to re-rate each indicator using the five-point Likert scale and give free comments on indicators.

After the second round, indicators were finalised. AHP consultation was then implemented to determine the weight of each indicator. AHP method was chosen in this study because it allowed identification of the relative importance of the five dimensions of service quality and their second-level indicators according to the opinions of experts. This may provide information for developing the service quality scoring system to assess the status of service quality and determine work priorities. We developed a AHP consultation questionnaire based on the identified indicators. Experts who returned the second-round questionnaires were instructed to weigh these indicators according to their experience and expertise [27]. The hierarchy structure is the basis to make judgement on the relative importance of one indicator over another. Pairwise comparison is applied to originate priority for all indicators. Pairs for each indicator to other indicators were set in the same level. Experts rated the importance of each indicator over other indicators using the Saaty 1–9 scale (1 = equal, 3 = moderate, 5 = strong, 7 = very strong, 9 = absolute and 2/4/6/8 = intermediate), and the comparison matrix was completed according to the value of relative importance [28]. For example, if the extent of importance of tangibility over reliability was moderate, then the entry of the matrix (tangibility, reliability) was 3 and the entry of the matrix (reliability, tangibility) was 1/3.

### Ethical considerations

The study protocol was approved by the ethics committee of the First Affiliated Hospital of Chongqing Medical University. Experts were assured of anonymity and confidentiality. All experts provided the informed consent.

### Data analysis

Data analysis was conducted using SPSS 25.0. Categorical data were presented by frequencies and percentages and continuous data by means and SD or medians and interquartile range (IQR). Mean and SD for each indicator were calculated to measure the central tendency and dispersion of the ratings, respectively. Kendall coefficient of concordance (Kendall’s W) and chi-square were used to assess the degree of agreement among experts. The Kendall’s W value ranges from 0 to 1, with a bigger value meaning a higher level of coordination among experts. *P* < 0.05 was considered statistically significant.

The AHP analysis included two phases. First, weight coefficients for the first-level and second-level indicators were calculated using the Saaty 1–9 scale to construct a pairwise comparison matrix. A higher weight coefficient indicates that the indicator is more important. Second, consistency test was conducted according to the calculated value, with lower consistency ratio (CR) indicating better coordination. CR value < 0.1 was considered acceptable.

## Results

### Characteristics of experts

Of the 40 experts initially contacted, 38 and 37 experts responded to the first and second round survey, respectively. The experts represented 23 institutions from the eastern, middle and western regions of China. The median years of experience were 22 years (range: 10–34 years) and 21 years (range: 10–34 years) for the first and second round, respectively. The characteristics of experts are shown in Table 1.

**Table 1:** Characteristics of experts

	Round 1 (*n* = 38)		Round 2 (*n* = 37)	
	*n*	%	*n*	%
**Age (years)**				
31–40	13	34.2	13	35.1
41–50	19	50.0	18	48.6
> 50	6	15.8	6	16.3
**Gender**				
Male	1	2.6	1	2.7
Female	37	97.4	36	97.3
**Education level**				
Bachelor	18	47.4	17	45.9
Master	11	28.9	11	29.7
PhD	9	23.7	9	24.4
**Years of experience**				
10–20	18	47.4	18	48.6
21–30	15	39.5	14	37.8
> 30	5	13.1	5	13.6
**Areas of expertise**				
Home care practices	10	26.3	10	27.1
Health care management	16	42.1	15	40.5
Home care research	12	31.6	12	32.4

### Round one

95.0% of experts responded in the first round. Consensus was reached for five first-level indicators: assurance (94.7%), reliability (92.1%), responsiveness (89.5%), tangibility (86.8%) and empathy (86.8%). Based on the consensus, seven (23.3%) second-level indicators were excluded: (1) visually appealing information materials; (2) showing interest in solving patients’ problems; (3) evident effect of service; (4) willingness to provide services to patients; (5) seeking to help patients; (6) getting enough support from institutions; and (7) being interested in doubts and suggestions of patients. For the remaining 23 second-level indicators, the level of agreement ranged from 81.6 to 94.7%, and mean scores and SDs ranged from 4.24 to 4.74 and from 0.55 to 0.79, respectively. The results of experts’ ratings on indicators in round one are shown in Table S1. Furthermore, adjustments to four (13.3%) second-level indicators were suggested by experts (Table 2). Thus, five first-level indicators and 23 second-level indicators were included in the second round.

**Table 2:** Results of experts rating on quality indicators for home care services in round two

Indicators	Agreement%	Mean ± SD
**1Tangibility**	89.2	4.48 ± 0.69
1.1 Convenient process to order services	94.6	4.68 ± 0.58
1.2 Detailed description of nursing services	91.9	4.65 ± 0.63
1.3 Transparent charges for nursing services	89.2	4.57 ± 0.69
1.4 Up-to-date equipment	86.5	4.49 ± 0.73
1.5 Nursing staff with dress code and work certificate ^a^	86.5	4.32 ± 0.71
**2 Reliability**	94.6	4.76 ± 0.55
2.1 Performing services right the first time ^a^	97.3	4.76 ± 0.49
2.2 Satisfying the needs of patients	91.9	4.51 ± 0.65
2.3 Providing services according to standards	89.2	4.65 ± 0.68
2.4 Providing error-free records for patients	94.6	4.49 ± 0.61
2.5 Providing services with adequate time allocated	94.6	4.54 ± 0.61
**3 Responsiveness**	91.9	4.59 ± 0.64
3.1 Communicating to patients about service provision	97.3	4.57 ± 0.55
3.2 Telling patients when the service will be provided	94.6	4.73 ± 0.56
3.3 Solving patients’ problems in a timely manner	91.9	4.46 ± 0.65
3.4 Considering patients’ complaints	94.6	4.49 ± 0.61
**4 Assurance**	97.3	4.73 ± 0.51
4.1 Nursing staff with adequate knowledge and techniques	97.3	4.81 ± 0.46
4.2 Being polite with patients	91.9	4.78 ± 0.58
4.3 Good communication with patients	94.6	4.73 ± 0.56
4.4 Answering patients’ questions carefully	89.2	4.59 ± 0.68
4.5 Feeling safe when using services	89.2	4.70 ± 0.66
**5 Empathy**	89.2	4.32 ± 0.67
5.1 Respecting and protecting patients’ privacy ^a^	89.2	4.70 ± 0.66
5.2 Offering emotional support ^a^	83.8	4.49 ± 0.77
5.3 Providing patients with individualised attention	94.6	4.51 ± 0.61
5.4 Understanding the specific needs of patients	91.9	4.43 ± 0.65

^a^ items that were modified; SD: standard deviations

### Round two

97.4% of experts responded in the second round. Consensus was reached on the five first-level and 23 second-level indicators: tangibility (5 indicators), reliability (5 indicators), assurance (5 indicators), responsiveness (4 indicators) and empathy (4 indicators). The level of agreement ranged from 89.2 to 97.3% for the first-level indicators and 83.8–97.3% for the second-level indicators. Mean scores for the second-level indicators ranged from 4.32 to 4.81 and SDs ranged from 0.46 to 0.77, indicating a more degree of consensus within the expert group. Table 2 presents the results of experts’ ratings on indicators in round two.

### Coordination of expert opinions

In round one, the Kendall’s W values were 0.47 and 0.28 for the first-level and second-level indicators, respectively (*p* < 0.001). In the second round, the Kendall’s W values were 0.54 and 0.40 for the first-level and second-level indicators (*p* < 0.001), which were more acceptable (Table 3).

**Table 3:** Coordination degree of expert opinions

	Kendall’s W	χ^2^	*p*
**Round 1**			
First-level indicators	0.47	89.25	< 0.001
Second-level indicators	0.28	313.49	< 0.001
**Round 2**			
First-level indicators	0.54	97.30	< 0.001
Second-level indicators	0.40	333.80	< 0.001

### Relative importance of indicators

Among the first-level indicators, reliability showed the highest weight (0.298), followed by assurance (0.237), responsiveness (0.182), tangibility (0.173) and empathy (0.110). CR value was 0.021 (< 0.1), indicating better coordination. In the second-level indicators, the weight coefficients ranged from 0.019 to 0.088, with CR values from 0.004 to 0.035 (< 0.1). Results of the weight coefficients for each indicator are presented in Table 4.

**Table 4:** Weights of quality indicators for home care services

Indicators		Weight	CR
First-level	1 Reliability	0.298	0.021
	2 Assurance	0.237	
	3 Responsiveness	0.182	
	4 Tangibility	0.173	
	5 Empathy	0.110	
Second-level	1.1 Convenient process to order services	0.050	0.006
	1.2 Detailed description of nursing services	0.042	
	1.4 Up-to-date equipment	0.033	
	1.3 Transparent charges for nursing services	0.031	
	1.5 Nursing staff with dress code and work certificate	0.019	
	2.1 Performing services right the first time	0.088	0.012
	2.3 Providing services according to standards	0.058	
	2.5 Providing services with adequate time allocated	0.055	
	2.2 Satisfying the needs of patients	0.051	
	2.4 Providing error-free records for patients	0.045	
	3.2 Telling patients when the service will be provided	0.049	0.022
	3.3 Solving patients’ problems in a timely manner	0.044	
	3.4 Considering patients’ complaints	0.044	
	3.1 Communicating to patients about service provision	0.043	
	4.1 Nursing staff with adequate knowledge and techniques	0.062	0.004
	4.2 Being polite with the patients	0.055	
	4.3 Good communication with patients	0.042	
	4.5 Feeling safe when using services for patients	0.040	
	4.4 Answering patients’ questions carefully	0.038	
	5.1 Respecting and protecting patients’ privacy	0.034	0.035
	5.2 Offering emotional support	0.026	
	5.3 Providing patients with individualized attention.	0.026	
	5.4 Understanding the specific needs of the patients	0.025	

CR: consistency ratio

## Discussion

With the increasing development of home care, the quality of care is required to be assessed and monitored. Based on the SERVQUAL model, a suite of 23 indicators assessing five dimensions of service quality in home care settings was developed. These indicators can be used to evaluate the service quality of home care from patients’ perspective and help institutions determining work priorities and improving the service quality in line with patients’ needs.

In this study, experts from different geographical contexts and the median length of professional experience suggested that experts represented a broad and experienced group. The response rates of the two round survey were more than 95%, indicating that experts showed more concern and continuing enthusiasm with this topic and considered quality indicators for home care services as an important issue. The coordination degree and consensus levels of indicators were increased in the second round, reflecting an increasing level of agreement on indicators among experts. Thus, the final indicators can be considered valid and useful to assess service quality of home care.

Indicators of the SERVQUAL model should be modified for different service environments and service subjects [16]. Quality indicators identified in this study suggested that experts in China rated good quality home care with the characteristics of reliability, assurance, responsiveness, tangibility and empathy. These indicators may reflect the needs of home care recipients. More specifically, for home care services, reliability included provision of correct services, standardised services, error-free records, adequate time allocation and satisfying patients’ needs. In terms of relative importance, reliability showed the highest weight. This indicated that experts considered reliability to be the most important aspect of good quality home care. Patients primarily concern with the improvement of their functional status and quality of life [9], and reliability is largely associated with service outcomes [19]. One review also suggested that patients perceived appropriate care with adequate time allocation to be a sign of quality care [29]. Current challenges for home care services include limited medical resources and equipment, time constraints, insufficient staff training and workforce shortages and instability [9, 22], which may be barriers to provide reliable services. Our study further highlights the importance of the reliability of home care services and more attention on strategies to ensure reliable services.

Assurance had the second weight, indicating a recognition that assurance is necessary to ensure the quality of home care. Patients usually perceived home care services provided by qualified and experienced staff to be good care [30]. Xia et al. also found nurse qualification as the primary factor of quality improvement of home care [31]. Home care needs of patients are often complex and vary significantly [2], and home care tasks are relatively independent and carried out with limited cooperation and consultation [32]. This calls for qualified home care nurses to provide care, such as sufficient knowledge and techniques, good communication skills and patience. Professional development needs of home care nurses should be addressed to support their continuing competence [33]. Home care nurses also expressed more training needs to update their knowledge and skills [2, 32]. However, most home care institutions lacked systematic training for nurses, especially in rural areas [34]. Therefore, identification of training priorities and provision of targeted training programs should be emphasized for home care nurses.

For home care recipients, it is important to receive timely care and understand the schedule for their care in advance [29]. Responsiveness dimension showed the third weight and included indicators related to provide timely service and consider patients’ complaints. The pressure of time is a common issue for home care nurses. Increased care complexity, staff shortages and heavy workload contribute to the inability of nurses to ensure timely service [33] and this appears to affect patients’ experience of service and quality of care [22]. Reasonable arrangement of service process and nursing stuff allocation is needed to provide timely and quality service. Additionally, patients’ satisfaction with home care quality may affect their health outcomes [31]. Home care services are usually performed in private homes without close supervision [2]. Complaints from patients should be resolved effectively to respect their rights and meet their needs and expectiatons.

In this study, tangibility dimension included process to order services, detailed description of care services, up-to-date equipment, charges of services and well-dressed staff, suggesting these indicators should also be targets for quality management for home care. One study revealed equipment with medication and supplies for first aid as an important factor of good quality home care [31]. Medical resources and equipment are critical to quality of home care. Limited medical resources and equipment were found common problems when providing home care [9]. Reasonable allocation of home care resources across geographical areas has been suggested to improve the balanced development of home care [34]. Furthermore, process of ordering care, descriptions and charges of services should be convenient to access for patients to facilitate their good service experience.

Providing home care services in patients’ own homes was described as a loss of privacy or autonomy and sufficient respect and empathy were expected from home care nurses [29, 30]. Though it ranked the least importance, empathy should be emphasised for an essential factor of good quality home care. Patients could show satisfaction with positive feedback from home care nurses [29]. According to the identified indicators of empathy dimension, nurses should give attention to the emotional demands from patients, understand their specific needs and provide care in a respectful way during care delivery. In addition, maintaining patients’ dignity and encouraging their independence were found to be important components of good quality care [22], supporting our indicators. The results suggested home care nurses should adequately respect and protect patients’ privacy to maintain their autonomy.

The significance of high-quality home care services to patient outcomes is currently under recognising. It is of great importance to develop indicators to measure the quality of home care services by means of a national consensus. In this way, this study adds to research on development of quality indicators for home care services from patients’ perspective. Although conducted in a Chinese healthcare context, there is potential for adaption in other healthcare settings. Identification of service quality indicators is helpful to tell the criteria for good quality home care and instruct home care institutions how to better provide home care services. Furthermore, these identified indicators can be used to assess and monitor the quality of home care services to better meet patients’ care needs and improve their health outcomes.

### Limitations

There are some limitations in this study. First, this Delphi process was undertaken in a Chinese context. Generalisation of our results to countries with different types of home care services should be attempted with caution. Second, instead of selecting experts using random sampling in an expert pool, experts were selected using purposive sampling, which may introduce a selection bias. Third, using expert ratings to derive indicators is highly dependent on the sample of experts and their understanding of the indicators. Although experts in this study were from different geographical contexts and with professional experience, the variation in expert ratings may influence the reliability of the identified indicators. In addition, the modified Delphi method did not provide opportunity for experts to make discussions on some indicators. Lastly, the implementation of quality indicators is important to confirm their practicability as well as their ability to discriminate between individual states. The subsequent procedure is needed to test the practicability and discriminatory capacity of these quality indicators in home care settings.

## Conclusion

Five first-level indicators and 23 second-level indicators for the quality of home care services have been identified via a national group of experts. Moreover, the reliability and assurance dimensions played the leading role in the quality of home care services. This suite of quality indicators is aimed at being used on home care recipients to help guide home care institutions to assess and monitor the quality of home care services and find areas that need to be improved. This can facilitate the improvement in home care quality. Future research is needed to validate these indicators in practice.

### Electronic supplementary material

Below is the link to the electronic supplementary material.

Supplementary Material 1

## Data Availability

All data generated or analysed during this study are included in this published article.
