# Supplementary material for: Identifying quality indicators for home care services: a modified Delphi and Analytic Hierarchy Process study
Source: BMC Nurs. 2024 Jul 19;23:494. doi: 10.1186/s12912-024-02169-4 (PMC11264849; doi:10.1186/s12912-024-02169-4)
Supplement: Supplementary file 1 — Supplementary Material 1 [file 12912_2024_2169_MOESM1_ESM.docx]

**Table S1 Results of experts rating on quality indicators for home care services in round one**

| **Indicators** | **Agreement%** | **Mean±SD** |
| --- | --- | --- |
| **1Tangibility** | 86.8 | 4.42±0.72 |
| 1.1 Convenient process to order services | 92.1 | 4.63±0.63 |
| 1.2 Detailed description of nursing services | 86.8 | 4.39±0.72 |
| 1.3 Transparent charges for nursing services | 86.8 | 4.39±0.72 |
| 1.4 Up-to-date equipment | 84.2 | 4.45±0.76 |
| 1.5 Nursing staff with uniforms | 81.6 | 4.29±0.77 |
| 1.6 Visually appealing information materials (i.e. pamphlets) ^a^ | 63.2 | 3.68±0.87 |
| **2 Reliability** | 92.1 | 4.68±0.62 |
| 2.1 Performing services right | 94.7 | 4.68±0.57 |
| 2.2 Satisfying the needs of patients | 89.5 | 4.50±0.69 |
| 2.3 Providing services according to standards | 86.8 | 4.55±0.72 |
| 2.4 Providing error-free records for patients | 89.5 | 4.45±0.69 |
| 2.5 Providing services with adequate time allocated | 92.1 | 4.48±0.65 |
| 2.6 Showing interest in solving patients’ problems ^a^ | 65.7 | 3.89±0.89 |
| 2.7 Evident effect of service ^a^ | 68.4 | 3.84±0.75 |
| **3 Responsiveness** | 89.5 | 4.53±0.69 |
| 3.1 Communicating to patients about service provision | 92.1 | 4.39±0.64 |
| 3.2 Telling patients when the service will be provided | 86.8 | 4.24±0.68 |
| 3.3 Solving patients’ problems in a timely manner | 86.8 | 4.45±0.72 |
| 3.4 Considering patients’ complaints | 89.5 | 4.29±0.65 |
| 3.5 Willingness to provide services to patients ^a^ | 68.4 | 3.79±0.78 |
| 3.6 Seeking to help patients ^a^ | 71.1 | 3.89±0.83 |
| **4 Assurance** | 94.7 | 4.68±0.57 |
| 4.1 Nursing staff with adequate knowledge and techniques | 94.7 | 4.74±0.55 |
| 4.2 Being polite with patients | 89.5 | 4.58±0.68 |
| 4.3 Good communication with patients | 89.5 | 4.68±0.66 |
| 4.4 Answering patients' questions carefully | 86.8 | 4.47±0.73 |
| 4.5 Feeling safe when using services | 84.2 | 4.50±0.76 |
| 4.6 Getting enough support from institutions ^a^ | 68.4 | 3.87±0.70 |
| **5 Empathy** | 86.8 | 4.26±0.69 |
| 5.1 Protecting patients’ privacy | 86.8 | 4.58±0.72 |
| 5.2 Listening to the patients and giving them comfort | 81.6 | 4.37±0.79 |
| 5.3 Providing patients with individualised attention | 89.5 | 4.42±0.68 |
| 5.4 Understanding the specific needs of patients | 89.5 | 4.34±0.67 |
| 5.5 To be interested in doubts and suggestions of patients ^a^ | 73.7 | 3.92±0.82 |

^a^ items that were excluded; SD: standard deviations
